# Foreign Bodies on the Iris

**Published:** 1879-11

**Authors:** Lucien Howe


### FOREIGN BODIES ON THE IRIS.

BY LUCIEN HOWE, M. D.

In cases where foreign substances are lodged on the interior of the eye, a serious question often arises as to the advisability of attempting their removal. The amount of injury produced, the size of the body, and the position where it has lodged, are indications sufficiently marked, in many instances, to determine at once the best course to be pursued.

There are cases, however, in which this is not so easily decided, and I would call attention to the fact, that an answer to the question, whether or not we should interfere, often depends upon the nature or composition of the foreign substance. This perhaps can be advantageously shown by two illustrative cases.

No. I. A little boy, D. L., came to me from Dunkirk, suffering from the effects of premature explosion of gunpowder. As a result, the entire upper part of his face was thickly studded with the imbedded particles, the left side being particularly disfigured. The lids were also swollen, lachrymation profuse, and photophobia extreme. An examination of the right eye, showed only a few grains in conjunctiva and cornea. But the injury to the left was more marked. The greater part of the cornea had been burned by the flash, causing the epithelial layer to become opaque. Numerous particles were deeply imbedded in that tissue, and some had evidently passed entirely through it, while others were thickly strewn upon the ocular portion of the conjunctiva. The patient was therefore etherized, and all the powder actually visible carefully removed. By means of anodynes the pain was alleviated, and under the use of atropine the condition of the eye greatly improved. As soon, however, as the opaque epithelium was replaced by a clear layer, a suspicious looking spot could be seen about the middle of the iris, in its superior and external quadrant. With the pupil dilated and the retracted iris thus thrown into folds, it was rather difficult to say whether this spot was a grain of powder, or simply a natural deposit of pigment. A solution of eserin was therefore dropped into the eye, which, by contracting the pupil, rendered the iris more tense and smooth, manifesting plainly the presence of a foreign body. Moreover, as the cornea continued to clear up, the line of entrance of the particle could be distinctly traced, and it soon began to make itself felt by the iritis which followed. This was sufficiently marked to cause some apprehension, but soon passed off, the injection disappeared and vision improved. Being obliged to leave the city about that time, I left the patient in charge of my friend, Dr. Abbott, and at the last visit the particle of powder was still distinctly visible, but the eye otherwise in a perfectly normal condition.

No. II. This patient, Peter S——, in June, 1878, while working at his trade as a stone cutter, was struck in the right eye by a particle of steel. At first it was hardly noticed, and not till some time afterward, did it give him any especial annoyance. When, at last, he applied for relief, there was found to be considerable ciliary injection, an exudation in the pupil, and swollen iris, while in the upper and external quadrant of that membrane a minute black point could be clearly discerned. The patient was at once urged to have the piece removed, but became alarmed at the suggestion and disappeared. However, on the 9th of Sept. following he returned, the symptoms, in the meanwhile, having become aggravated, and the vision so impaired that he could not count fingers across the room, or $V=\frac{3}{100}$. He was then ready to submit to operation. Accordingly, chloroform was administered by Dr. Macniel, and after making a wide incision at the corneal margin, like that required for an iridectomy, I passed a pair of fine forceps into the anterior chamber, and attempted to extract the steel. The adhesions in the vicinity made its detachment impossible, and I therefore drew out with it a considerable piece of iris. This was cut off and the remainder being returned to its proper place, the eye was closed with a bandage. The wound healed rapidly, the improvement beginning with the subsidence of the inflammation, and fifteen days afterward, the vision had increased almost twenty fold—more exactly $V=\frac{20}{30}$. At his last visit, on the 16th of October, 1878, the injection had entirely subsided, and the patient was much gratified at the result.

Here, then, we have two excellent cases for comparison. In both the foreign substance was about the same size, and lodged on the same part of the same structure. In one case, its presence produced little or no inconvenience; in the other, an inflammatory process, with failure of vision resulted, which symptoms disappeared on the removal of the foreign body. The only well marked difference was in the composition of the substances—one mainly made up of carbon, the other was steel—the first, liable to decomposition only in a slight degree; the second, readily oxidizable.

I would not pretend to say how or why it is, that this destructive process, in an almost microscopic particle, can so effect the nerves and vessels in its vicinity as to produce a well marked inflammatory condition.

Clinical observations, however, tend to establish the fact, and in my experience, at least, it has occurred that grains of gunpowder, fragments of coal, glass and stone, have produced, in general, less irritation in the eye than substances more easily decomposed. This difference in behavior would appear to be an important point, and one usually omitted in the text books. It should not be supposed, however, that even the most unchangeable of foreign bodies can remain in the eye without considerable danger of subsequent inflammation. The tendency to sympathetic iritis is so great, and its results so serious, that every case of the sort must be looked upon with suspicion. It is only a matter of some satisfaction to think that when the substance is one not easily decomposed, the prospects are better for a favorable result. Nor would I be understood as intimating that the two cases cited constitute of themselves sufficient basis for any valid conclusion. They simply illustrate a series of facts quite frequently observed, and as such their history, and the inference to be derived from them, appear to be worthy of record.

